# Performance of QR Code Detectors near Nyquist Limits

**DOI:** 10.3390/s22197230

**Published:** 2022-09-23

**Authors:** Przemysław Skurowski, Karolina Nurzyńska, Magdalena Pawlyta, Krzysztof A. Cyran

**Affiliations:** 1 Department of Computer Graphics, Vision and Digital Systems, Silesian University of Technology, Akademicka 16, 44-100 Gliwice, Poland; 2 Department of Algorithmics and Software, Silesian University of Technology, Akademicka 16, 44-100 Gliwice, Poland

**Keywords:** QR code, augmented reality, Nyquist limit, simulation

## Abstract

For the interacting with real world, augmented reality devices need lightweight yet reliable methods for recognition and identification of physical objects. In that regard, promising possibilities are offered by supporting computer vision with 2D barcode tags. These tags, as high contrast and visually well-defined objects, can be used for finding fiducial points in the space or to identify physical items. Currently, QR code readers have certain demands towards the size and visibility of the codes. However, the increase of resolution of built-in cameras makes it possible to identify smaller QR codes in the scene. On the other hand, growing resolutions cause the increase to the computational effort of tag location. Therefore, resolution reduction in decoders is a common trade-off between processing time and recognition capabilities. In this article, we propose the simulation method of QR codes scanning near limits that stem from Shannon’s theorem. We analyze the efficiency of three publicly available decoders versus different size-to-sampling ratios (scales) and MTF characteristics of the image capture subsystem. The MTF we used is based on the characteristics of real devices, and it was modeled using Gaussian low-pass filtering. We tested two tasks—decoding and locating-and-decoding. The findings of the work are several-fold. Among others, we identified that, for practical decoding, the QR-code module should be no smaller than 3–3.5 pixels, regardless of MTF characteristics. We confirmed the superiority of Zbar in practical tasks and the worst recognition capabilities of OpenCV. On the other hand, we identified that, for borderline cases, or even below Nyquist limit where the other decoders fail, OpenCV is still capable of decoding some information.

## 1. Introduction

Image and object recognition methods are important research and engineering areas. They are of special priority for computer systems operating with real (physical) objects that are not directly connected. In applications such as augmented reality (AR) [1,2,3] or surveillance [4,5], identification of relatively small objects in broad scenes is an important factor for their efficiency. The rapid development of novel CV methods, such as deep learning, has opened brand new capabilities to the CV area. These methods are now available even to the relatively small devices used in AR. However, despite the continuous hardware development, there is still a gap between devices and PCs in computing capabilities, which results in limited throughput of modern deep learning methods [6]. It makes them hardly usable for real-life applications, where response time is one of key requirements.

QR codes (and other codes) have been present in AR for quite a long time. They bring supplementary information to the scene by visual means, and they are applied in learning, advertising, and exhibition [7]. The motivation for bringing supplementary visual information into AR for serious applications might be connected to the Industry 4.0 concept and is rather clear [1,2]. Tasks such as manufacturing, inspection, maintenance, or diagnostics of technical facilities require recognizing numerous objects, which might be cumbersome to identify just by means of computer vision. These objects could be virtually identical, or they can occur in numerous instances. Moreover, the complete inventory of objects to recognize in the scene could be uncertain, or objects of the same class could visually appear differently depending on the vendor. The solution here is that each object brings not just an identifier, but the information necessary to identify it. One of such techniques, mature and widely adopted, are barcodes, of which QR codes are probably the most popular variant.

**Figure 1:**
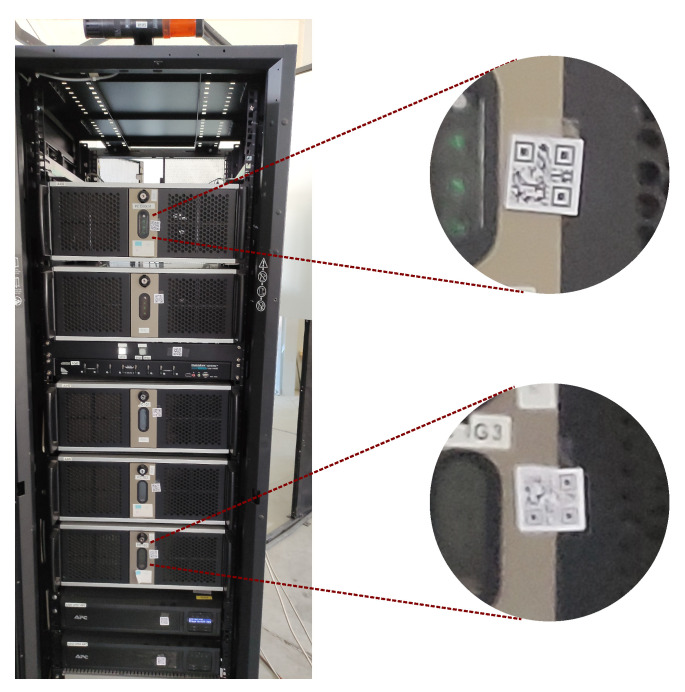
Exemplary situation illustrating small QR codes in inspection of rack with numerous PCs. Picture taken with Microsoft HoloLens 2 front camera at its full photographic resolution.

Currently, QR code readers perform well with decoding codes of good visibility—large, near, or both. However, they do not perform so well if the codes are small, blurred, low contrast, or poorly visible as being just minor parts of the scene, and the decoders vary in their decoding abilities. An example of a difficult situation is demonstrated in Figure 1, where a rack of PCs is inspected using an AR device from approximately 3–4 m distance. In this situation, we have numerous instances of computers that can be distinguished just by the identifiers, such as QR codes. Unfortunately, none of the codes is readable using any of the readers used in this research; the decoding issues are demonstrated in zoomed parts—the upper one illustrates small module size (2–3 pixels), whereas the lower is additionally out of focus because autofocus was matched according to the upper contents of image. Moreover, according to the best practices in Microsoft guidelines [8], such a size of QR codes is too small for system-assisted QR code decoding.

The distortions which can occur in the real imaging system are: noise, autofocus (AF) issues, uneven lightning, geometric distortions, QR code damage, and others. Typically, testing the QR code decoders requires having a prepared test dataset (e.g., [9]) representing considered distortions. Such databases, containing a lot of examples, do their job, but they have also limitations—we cannot control the distortion degree. Additionally, it is difficult to create a database of images containing numerous small and tiny QR codes, that would not be affected by AF blurring or noise.

The main goal of this research is to analyze the limits of the abilities of QR code readers, coming from the imaging system limitations and sampling theory. In the article, we propose a software-based method for evaluation of the QR code decoders for application in borderline cases. In such cases, QR codes are small, and their modules are of sizes comparable to the camera pixels. Thanks to in-memory noiseless processing, we were able to restrain the other factors from affecting the results.

The contribution in performance analysis is twofold. First, we analyze the selected QR code decoders for their timing performance and recognition ratio at the near Nyquist recognition limits. We analyze the recognition abilities with respect to the actual resolution capabilities of a hardware, modeled with a modulation transfer function (MTF). Next, we analyze the recognition ratio, additionally taking into consideration the size of the image into which the QR code is placed.

The article is organized as follows: Section 2 describes the background; Section 3 describes the experiments; Section 4 presents the results and their interpretation; and Section 5 provides the summary and conclusions.

## 2. Background

### 2.1. QR Codes

Barcode systems [7] are a class of methods intended to bring supplementary information into the scene through visual means. They are intended for robust and flexible delivery of arbitrary contents decoded by machine in a single readout.

**Figure 2:**
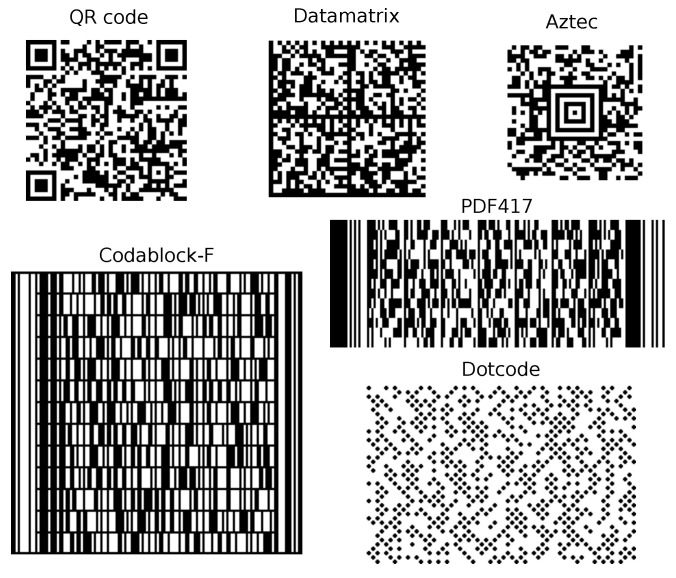
Exemplary 2D barcodes containing the same text comprising 89 characters.

The barcodes (see Figure 2), such as QR code [10], are intended to deliver virtually any content. Their design is mainly focused on the robustness and usually does not impose difficult real-time requirements, since the typical usage is a single barcode at a time. The maximal code capacity is of an order of kilobytes, and its flexibility is achieved by code spatial size scaling. Furthermore, the barcodes offer increased robustness due to usage of error correction codes, such as Reed–Solomon codes in QR codes and Datamatrix.

Initially, QR codes were designed for purposes of automotive industry manufacturing [10]; however, they became adopted to numerous other applications such as marketing, shopping, storing, and others [11]. Their widespread use can be associated to camera equipped mobile devices [12].

**Figure 3:**
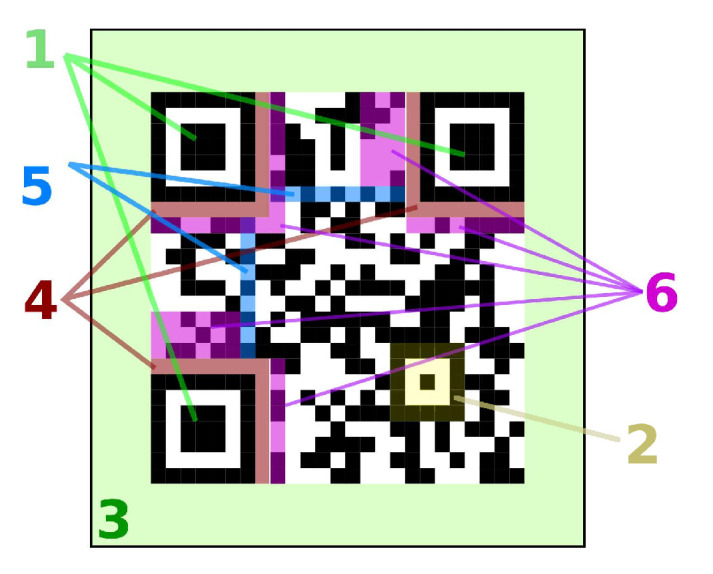
Topology of a QR code.

The QR code [10] is a rectangular 2D binary code containing several predefined features, as depicted in Figure 3. Its size depends on features such as capacity and level of correction through Reed–Solomon error correction bits, but foremost the physical size depends on the size of the module, which is the unit size for all the other components, and actually is a visual representation of a single bit. Basic QR code topology is comprised as follows:

1. Finder pattern (FIP)—exactly three predefined patterns (or the single one in micro QR).
2. Align—zero to many additional patterns for orientation identification.
3. Quiet zone.
4. Margins.
5. Timing—syncing pattern of interleaved 0 and 1’s.
6. Information fields such as format and version.

The amount of information the QR code can carry depends on the QR code size and correction level. The rectangular side sizes are defined with the code version number between 1 and 40, where the size is related as:

size=4×versionNumber+17,

therefore, the possible sizes vary between 21 and 177. The code capacity is between 55 and 3916 bytes approximately, and varies due to presence of control elements. Additionally, the final capacity of a QR code is also limited by the amount of error correction codes (ECC). They are specified by the correction level, defining the capability (percentage) for error correction percent. However, Reed–Solomon codes have *t* correcting bits and are capable of identifying up to *t* errors and correct up to t/2 of these. The QR code standard defines four levels of correction:

- L (low)—able to correct up to 7% loss.
- M (medium)—able to correct up to 15% loss.
- Q (quartile)—able to correct up to 25% loss.
- H (high)—able to correct up to 30% loss.

### 2.2. Sampling Limits

Image sampling follows Shannon’s sampling theorem [13], which states that for the perfect reconstruction, the minimal sampling frequency should be double the maximal frequency present in the signal—Nyquist limit (Nyquist frequency is in fact the reverse of this statement: maximal reproduced frequency is half of the sampling frequency). However, in the case of QR codes, the term signal frequency can be understood in two ways: first, considering it as a frequency carrying code modules as samples and second as frequencies representing contents of the image.

Of these two, we would consider the former one as the actual one, where the pixels to QR code modules are related 1:1, since it defines the limiting frequency for the useful content. Considering visual representation, the barcode images contain square patterns that result in infinite frequency components in Fourier spectral domain, so they might cause aliasing during sampling very easily. However, we would consider it rather as representation artifacts, not the information.

Another issue with sampling near the Nyquist limit is the fact that it is susceptible to the subtle subpixel shifts (phase), which averages the contents between pixels and clutters the visual contents depending on the shift.

**Figure 4:**
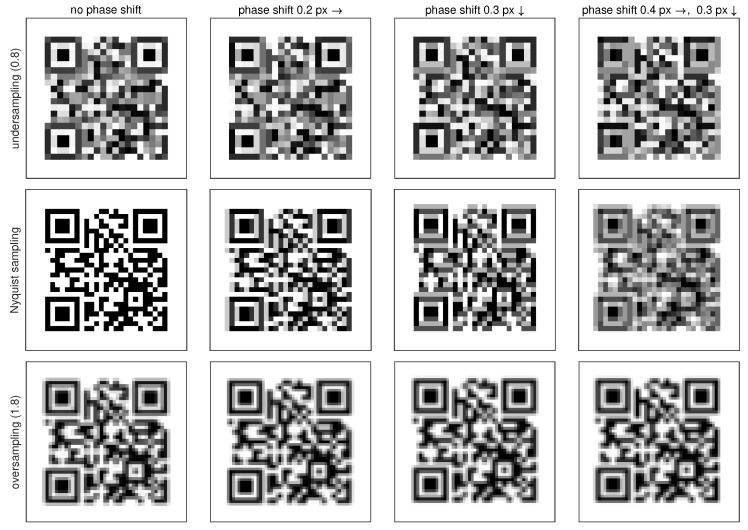
Sampling QR code near the Nyquist limit.

All these are illustrated in Figure 4, where the upper row shows aliased, undersampled contents; in the middle, we demonstrate QR code sampled at Nyquist limit; and at the bottom we see slightly oversampled QR code. All these are demonstrated at various subpixel shifts.

### 2.3. Resolution Capabilities in Digital Imaging

**Figure 5:**
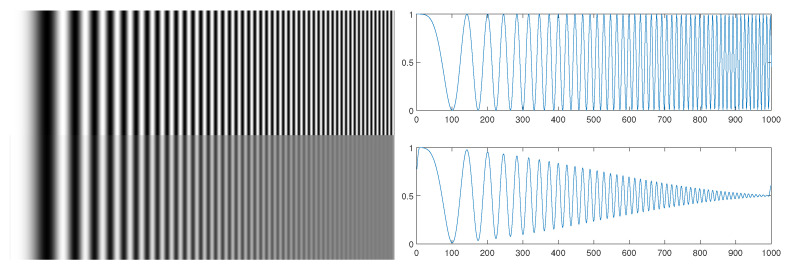
The basis for MTF concept: sine wave degradation as image and plot.

Shannon sampling theorem describes the resolution abilities of any digital imaging system at the very basic level. While the Nyquist limit defines the maximal theoretically reproducible frequency, the frequencies below that limit are also affected by the physical limitations of the imaging system. It attenuates the higher parts of available spectrum of the image capture system, which results in sharpness decrease. The blurring process can be described in terms of signal processing [14] as a point spread function (PSF), or in a more convenient way, using its spectral form—modulation transfer function (MTF). The imaging system is considered as an optical filter, where amplitude transmission ratio of certain frequencies is represented by appropriate MTF values which combine low-pass characteristics and mismatch between signal and sensor grid [15]. The idea is depicted in Figure 5, where the MTF can be visually identified as an envelope of decaying signal. Considering the sine wave model, MTF is defined as:

(1) $$\mathrm{MTF}\left(f\right)=\frac{V_{max}^{out}\left(f\right)-V_{min}^{out}\left(f\right)}{V_{max}^{in}\left(f\right)-V_{min}^{in}\left(f\right)}$$

where: *f* is the frequency (associated with location in sine wave image) and *V* is the image brightness value.

**Figure 6:**
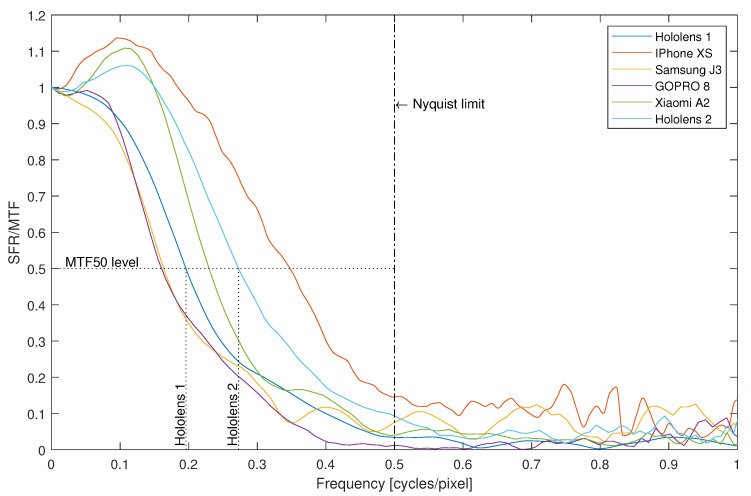
MTF values for exemplary devices, with depicted MTF50 values for HoloLens devices.

On the basis of MTF, a few summarizing scalar measures were proposed for simple comparison of imaging system performance—MTF*q* such as MTF30, MTF50, and MTF70. They describe at which relative frequency the MTF reaches a certain fraction (percent) of transmission—MTF50 is a common one, using 50% of transmitted level, and it is used in further parts of this research. In Figure 6, an example of obtaining MTF50s values is also illustrated for HoloLens 1 and HoloLens 2.

Various methods were proposed for MTF estimation. The slanted edge method [16] is the most popular and mature one (ISO 12233); hence, we employed it in this research. It employs superresolution techniques, so it is capable of characterizing the transmittance beyond the Nyquist limit. We obtained several illustrative MTFs for different devices which could be used for AR purposes—these are demonstrated in Figure 6. It clearly shows that acquisition abilities decrease with increasing frequencies. However, in the case of three devices, low frequencies were boosted above 1 because of software image sharpening.

### 2.4. QR Code Detectors

The recognition of QR code involves two key stages—tag discovery through FIP localization and orientation estimation and decoding. We could distinguish two main approaches for the QR-code localization: image based and line scan based.

**Figure 7:**
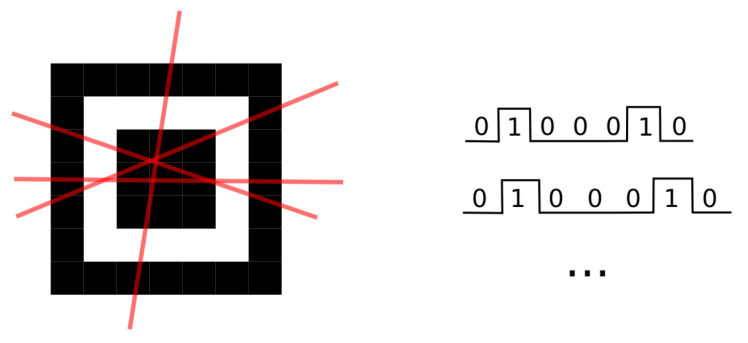
Finder pattern line scans and two exemplary profiles of 1:1:3:1:1 proportions (dark/bright/dark/bright/dark) depending on the line scan angle.

The image-based approaches locate the tag using 2D pattern matching for QR code localization, with methods such as mathematical morphology [17], histogram matching [18], Viola–Jones [19], or deep learning neural networks [20]. However, the line scan–based approaches [10], which mimic the behavior of the laser scanners of barcodes, are the most popular ones. Decoders such as ZBar [21], ZXing [22], and OpenCV [23] all employ the line scanning approach, where the image is scanned pixel by pixel, looking for the FIP patterns in rows and columns (1:1:3:1:1) as shown in Figure 7. Three detected FIPs allow identifying orientation of the code in the scene. Further improvements to the basic line-scan approach involves compensation of geometric distortions and partially corrupted finder patterns [24].

QR code locating is a critical stage for performance. Locating the FIPs in the scene is a computationally lightweight task; however, the increasing dimensions of images in modern devices makes it more and more time-consuming. For that sake, some detectors scale down the image, making the processing faster, at the cost of reduced detection abilities.

## 3. Materials and Methods

### 3.1. Outline

The problem with QR code detection is two-fold. First, the computation time increases with the size of input image. Second is the dependence of the recognition rate on numerous factors, such as: barcode orientation and scale, image blurring during acquisition, surrounding image, and image preprocessing in QR code scanner. Therefore, we assumed to perform numerical simulations to discover how they affect the performance of the QR readers.

### 3.2. Testing Software and Hardware

In the analysis step, we employed three up-to-date and freely available QR code scanners—ZXing, ZBar, and a detector that is part of OpenCV. They share the basic line scan approach; however, they differ in implementation details. Our test bed was a Dell Latitude E5440 laptop equipped with 16GB RAM, Intel i5-4300U CPU operating at 2.5 GHz, and GPU support was not used. It adheres quite well to the processing capabilities of contemporary AR devices such as smartphones and AR glasses like HoloLens (with no computational hardware support for user provided CV), since they offer kind of a trade-off between the performance and power consumption.

### 3.3. Scenarios and Measures

During the experiments, we focused on the two test cases involving the QR decoder:

- Simple decoding—this involved the simplest images of just the QR code so it revealed the pure recognition capabilities and performance of decoder, since the overhead to locate QR code was negligible;
- Locate-and-decode— this was focused mainly on QR code locating; we inserted QR codes at random positions into a relatively large background image, so the results were influenced by both the locating and decoding stages in the experiment.

The main performance criterion, recognition ratio, was measured in all the cases and parameters. Additionally, average processing time was also measured. The experiments involved numerical simulation of a QR code image, inpainted into an image with different pixel shifts and rotations with subpixel accuracy (using linear interpolation) to simulate only very basic geometric transformations. For every single QR code, it results in numerous image realizations to be recognized—the recognition ratio is therefore defined as the number of correctly identified *C* and recognized realizations to their overall number *N*:

(2) $$RR=\frac{C}{N}.$$

**Figure 8:**
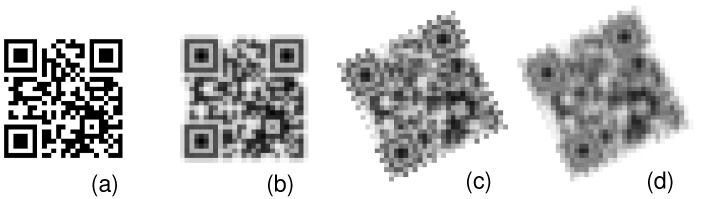
Example of MTF-based simulation of distortions during acquisition of QR code at Nyquist limit: (**a**) perfect ‘hard pixel’, (**b**) blurring due to Gaussian low-pass, plus blurring due subpixel misregistration because of: (**c**) rotation, (**d**) plus subpixel shifts.

Resolution capabilities were tested for the scale between 1 and 7 of the QR code module; hence, the basic unit component width varied between 1 and 7 pixels. These values are spanned between Nyquist limit and the size, which we preliminarily identified as being detectable in most situations. The MTF-based blur simulation was adjusted to reflect the intended MTF50 value in a two-step process. First, with use of Gaussian filter, we simulated low-pass filtering in optics. Next, using numerous interpolations, we simulated phase shifts (of equal probabilities), which on average contribute to the overall MTF with sin(x)/x component [15]. In order to keep control of the process, we used lookup table (LUT) to match between intended MTF50 and Gaussian dispersion + phase shifts. LUT was precomputed for varying width of Gaussian blur for which MTF50 was estimated using slanted edge method. These were calculated for MTF50 in range between almost complete information loss (0.02) and above the values obtained by the best of the devices we tested (0.5) with 0.005 step. In most of the experiments, we used Version 2 (25 × 25) QR code with M level of error correction code. The main steps of simulation of the sampling based distortion involving MTF, which were applied to this QR code, are depicted in Figure 8.

#### 3.3.1. Decoding Performance

The first experimental scenario is intended to identify the upper limits and minimal times for the QR code reading in both analyzed scanners. The images contained only the QR code without any additional margins other than resulting from rotation and specifications (quiet zone). The QR code images were blurred according to the MTF50 within the assumed range, and the scale swept the assumed range.

For every scale (from 1 to 7 with 0.25 pixel step) and MTF50 (between 0.02 and 0.5) we obtain RR on the basis of the number of realizations of MTF based distortions. For that purpose, additional image transformations were applied to the scaled and preliminary blurred image to get in total the intended MTF. They are subpixel phase shifts between −0.5 and 0.5 with 0.1 pixel step and rotations between 0 deg and 90 deg with 5 deg steps. It resulted in 2299 realizations of an image to recognize, which are the basis to compute recognition ratio.

**Figure 9:**
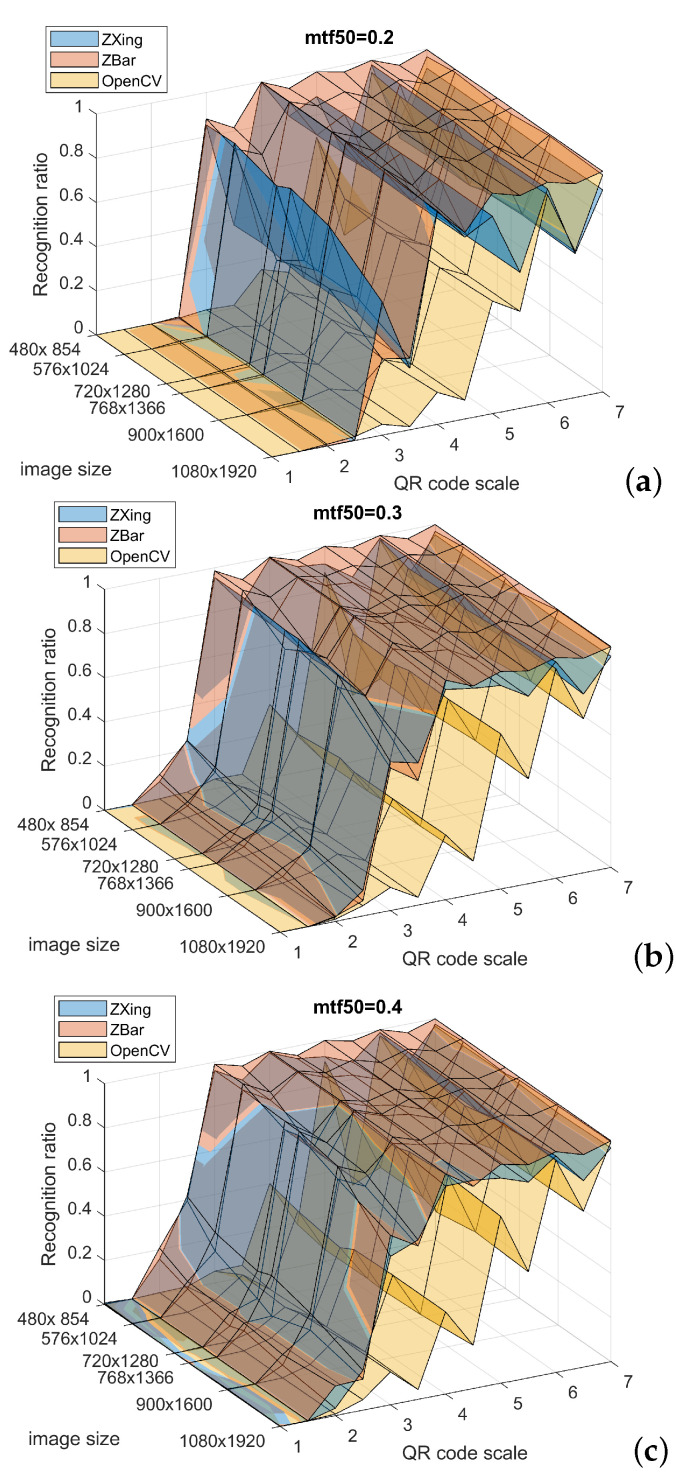
Recognition ratio for detect and decode for: (**a**) MTF50 = 0.2, (**b**) MTF50 = 0.3, (**c**) MTF50 = 0.4.

After observing the results of extensive simulation for various MTF50 and scales, we decided to perform an in-depth simulation at the Nyquist limit (scale = 1)—above and below. Since the initial observations (Figure 9) suggested that even at Nyquist limit, it is possible to decode some information, we decided to check how it looks depending on the error correction code levels. For that purpose, we performed inquisitive simulations as previously (phase shifts, rotations, and MTF50 ranges), but at selected scales (0.95, 0.98, 1, 1.05) and additionally used all four ECC levels (L, M, Q, H). Here we employed a simple version 1 QR code (25 × 25), containing just 3 letters.

#### 3.3.2. Detect-and-Decode Performance

**Figure 10:**
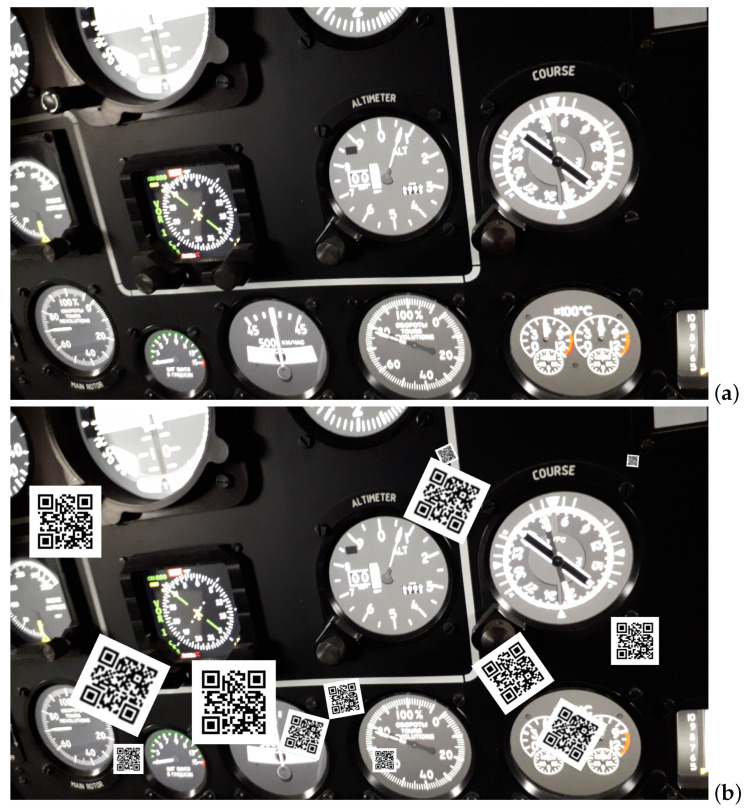
(**a**) Background image for the detect-and-decode scenario in maximal size (1920 × 1080); (**b**) background image inpainted with QR codes (at MTF50 = 0.4) at all sales used in the research in random locations and rotations (note that, in experiments, they occur one at a time).

The detect-and-decode test case resembles the previous one significantly. Three differences differentiate the case from the previous one. The first difference is another series of scales: now they are calculated from 1 to 7 with 0.5 pixel step. The second difference to the first experiment is assuming just 3 MTF-based blurring levels, which are represented by MTF50 = 0.2, 0.3, and 0.4, respectively; this adheres to the results presented in Figure 6. The key difference is in the image generation for the test. The QR code (actually each of its 2299 realizations) is placed in the background image sequentially in 30 randomly drawn locations; hence, the averaged recognition ratio computation is based on a notably larger number of recognition tasks (2299 × 30). The image presents technical equipment, cockpit of helicopter simulator (see Figure 10), which was selected for two reasons. First, it is an environment difficult for the decoders: it contains numerous contrasts, high dynamic of lightness and high number of details at different scales, including relatively small line patterns. Second, it is related to our current project on the application of AR goggles in a flight simulator scene.

## 4. Results and Discussion

### 4.1. Decoding Performance

**Figure 11:**
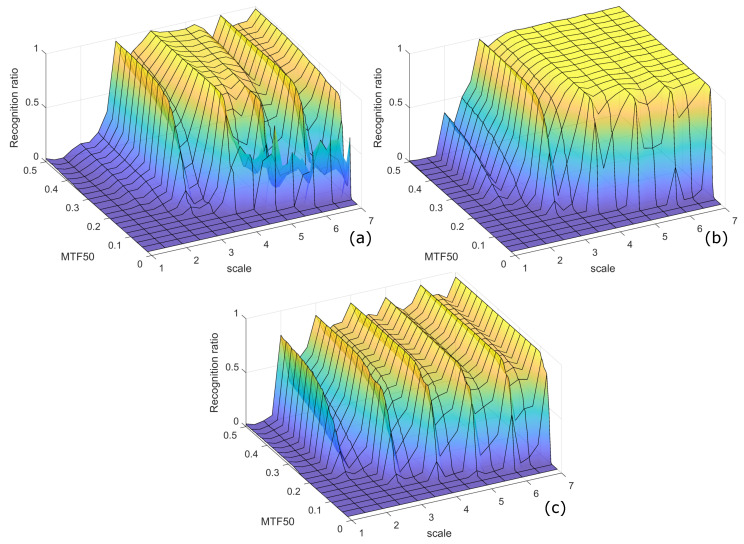
Recognition ratio for QR code image decoding for: (**a**) ZXing, (**b**) ZBar, (**c**) OpenCV.

**Figure 12:**
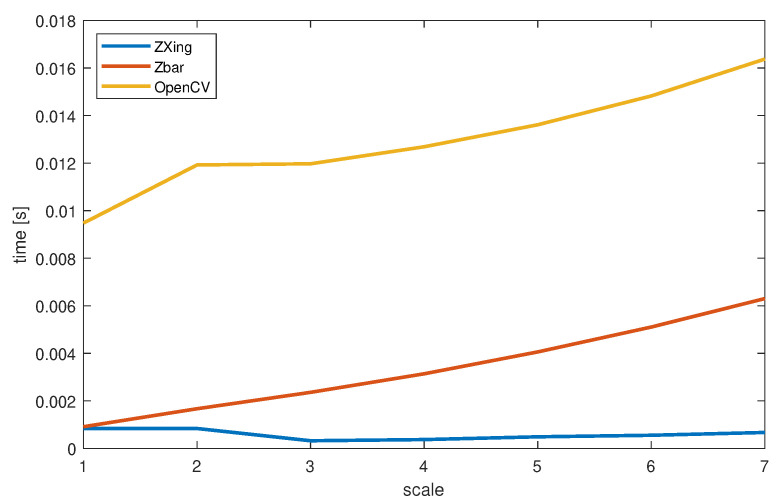
Recognition time for the simple QR code decoding for the two QR code readers.

The recognition ratio depending on scale and MTF50 is demonstrated in Figure 11. The computation time was not affected by the QR code blurring image (represented by MTF50); hence, it is presented in Figure 12 as a simple 2D plot of scale versus averaged execution times.

**Figure 13:**
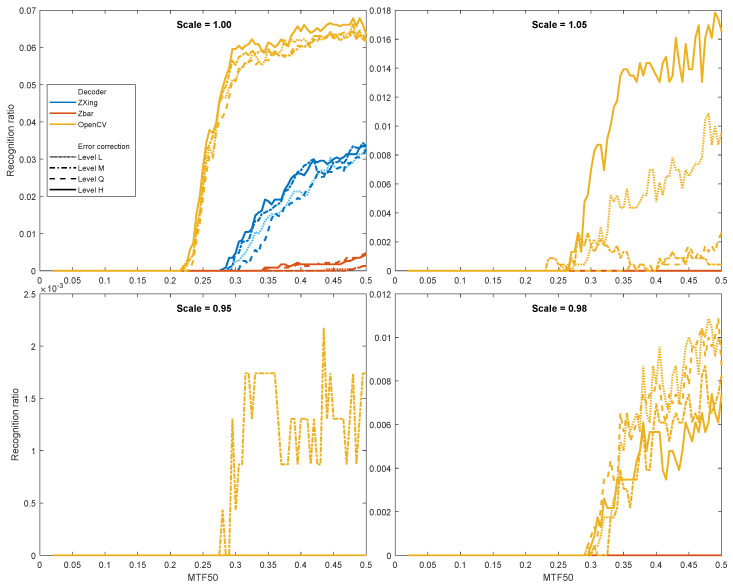
Recognition ratio of QR code decoding near the Nyquist (Scale = 1) limit. (The blue and red lines that ‘disappeared’ in three subplots are actually there, but at zero level).

The results of an additional in-depth simulation at the Nyquist and near the limit (above and below) is included in Figure 13. Please note the variable vertical scales when comparing the subplots.

Regarding the recognition abilities, the extensive review of scales and MTF50 is presented in Figure 11. The performance, measured using, varies between the decoders, despite the fact that all the three share the same (line scan) method of locating the QR codes. Not surprisingly, the larger the scale of the QR code and the higher the MTF50, the better the outcomes. However, there are some less obvious observations. For clarity, we summarize them as follows:

- It is clearly visible that all detectors achieve high performance with RR≈0.9 or more for the QR codes with module size of 3 pixels and above, with some advantage of ZBar over its counterparts.
- Despite common belief that ZBar outperforms the other decoders in QR code decoding, we could identify cases when ZXing and OpenCV brought higher RR. It is so for very small QR codes, where the module size is between 1 and 3 pixels.
- A noteworthy degradation is observed for partial (fractional) scales, when a single source pixel matches a non-integer number of pixels. It is especially visible for OpenCV and ZXing as a repetitive pattern along the scale axis, while ZBar is affected to lesser extent.

Probably, the latter fills the gap between simulation and real life image acquisition. It is highly unlikely that a QR code would be registered by a perfectly matched sensor grid, so those repeating peak values in OpenCV results, along the integer axis, should be considered as theoretical, not actual performance.

An insight into sampling near the Nyquist limit, presented in Figure 13, discloses how resilient the QR codes are to poor sampling, and the capabilities of ECC to compensate it. The detailed observations are as follows:

- The Nyquist limit (Scale = 1) is generally a difficult situation for any of the decoders; however, they still are able to decode some information. The best results are offered by OpenCV, whereas ZBar returned the poorest results in this case. It conforms also to the results in Figure 11, where the OpenCV offers the best RR for low scales.
- MTF50 = 0.25 can be considered as the boundary value in the case. Below this, we obtain virtually no positive results.
- Results using various error correction codes are a bit ambiguous. Below Nyquist, only OpenCV is still able to decode little information; the deeper below the Nyquist limit, the lower the results we obtain. A noteworthy fact is that lower levels (L and M) offered the best performance at scale = 0.98, and the Q level was the only case resulting in a few non-zero for scale = 0.95.
- Slightly above the Nyquist (Scale = 1.05), where QR code modules occupy slightly more than one pixel (a thus are dispersed among them), low (L), and, especially, high (H) levels of ECC ensured smaller information loss due to inter-pixel dispersion of modules.

Additional timing analysis (Figure 12) shows, for the first test scenario, that the simple identification and decoding of the QR code is a lightweight task, and its execution time increases slightly with increasing scale of the QR code. It could be performed between ≈60 and 200 codes per second on a relatively low-end hardware. The ZBar and OpenCV are on par, whereas ZXing offers notably faster performance.

### 4.2. Detect-and-Decode Performance

**Figure 14:**
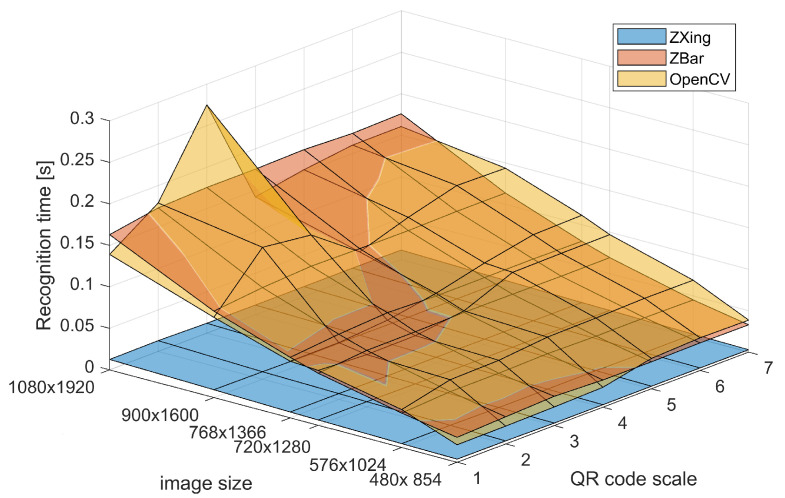
Recognition time for detect-and-decode of QR code in images of various size.

Averaged recognition ratios for all random locations are demonstrated in Figure 9. Average computation times are demonstrated in Figure 14. Note that image sizes in this plot are labeled using image dimensions (W × H), but scalar numbers representing location on the axis are according to the diagonal length of respective dimensions. Also note that the image size axis is inverted between these images for the clarity of results.

The placement of QR code in the image is a factor that might have influence on the decoding abilities. Before the decoding, the code must be located within the image by the decoder. That task might be slow in large images; therefore, the typical strategy is to reduce the resolution of the image. Browsing the source code, we identified that OpenCV QR code detector (https://github.com/opencv/opencv/blob/master/modules/objdetect/src/qrcode.cpp (accessed on 15 September 2021)), in an entry stage, resizes the image proportionally making its smaller dimension to be always 512 pixels. Similarly, ZBar (http://zbar.sourceforge.net/iphone/sdkdoc/optimizing.html#resolution (accessed on 15 September 2021)) enforces the larger dimension to be smaller than 640.

The experimental observations, based on Figure 9, are summarized in following points:

- For small scale QR codes (Scale = 1, 1.5) the RR is close to zero, for all decoders and all MTF50 values, the decoders start to recognize anything at Scale = 2.
- Partial (rational) scales of QR code result in degraded RR—the same as in the simple decoding task, and the decoders are affected to different extent.
- ZXing and ZBar return quite similar results, whereas OpenCV in general returns notably worse outcomes.
- For the scale ≥ 3.5 ZBar reaches about 0.9, ZXing offers similar but slightly worse performance at such scales; it can be especially observed for lower MTF50 = 0.2.
- The size of image affects mainly at lower scales: 3 and below.

Considering the execution time of the detection-and-decoding process (Figure 14), it is clearly more burdensome than simple decoding. It requires locating the QR code within the image; hence, for large images it could be as low as 5 frames per second (FPS) on the test hardware for ZBar or OpenCV. However, reducing the resolution could improve this value to 20 FPS, or even less if using ZXing. The scale of the QR code placed into the image is notably less important to the processing time than the overall size of image.

## 5. Summary and Conclusions

In the article, we proposed and verified a software method for evaluation of the QR code decoder performance. Thanks to avoiding physical representation, we were able to perform the tests in borderline cases, considering only images in proximity of the Nyquist and incorporating only those distortions which are inherently related to sampling (PSF/MTF).

Of course, one might consider such results to be a bit too optimistic, yet they show the limits in performance for the QR code readers. Moreover, the influence of other challenges, such as color filter array de-mosaicing, noise, autofocus issues, poor lighting, and others that could occur in real life; they can be easily incorporated into the software model using image preprocessing such as image blurring or contrast limiting, and could be a topic of further research.

A corollary to the problem discussed in this work is the question of how the geometric setup of a scene influences the performance of QR code detectors. It could be modeled within the proposed framework, employing 2D perspective transformation; however, the transformation matrix parameters (4) are not very intuitive, so controlling the experiment and interpreting the results would be cumbersome. It seems that an alternative approach, a 3D projection model of QR code acquisition from a distance, is better suited for the perspective distortion model. It describes the scene with parameters such as observation position and angle, which would better adhere to everyday experience.

The practical conclusion of this research partially confirms the common belief that the ZBar outperforms the other detectors, since it offers higher recognition rate. However, not in every case, for QR codes very close to the Nyquist limit, OpenCV outperforms the other tested decoders. Our results can be combined with QR code segmentation for improving detection efficiency. After identification of the QR code area, one could estimate the size of module scale using e.g., autocorrelation or Fourier transform and then use a decoder appropriate to the scale.

In conclusion, in the tests, we identified upper limits for performance of QR code detectors. Additionally, we identified the ability of QR codes to overcome Nyquist limit (not much, but still) with error correction codes. Moreover, we could draw some useful conclusions, such as minimal usable scale of QR code, or which decoder for which scale results in better recognition.

## Data Availability

Not applicable.

## Abbreviations

The following abbreviations are used in this manuscript:

ECC	Error Correction Code
LUT	Look-up table
MTF	Modulation Transfer Function
PSF	Point Spread Function
QR code	Quick Response code
RR	Recognition Ratio
